# Impact of Anemia on Outcomes Following Total Hip Replacement Surgery and Patient Prognosis

**DOI:** 10.1111/os.70208

**Published:** 2025-12-12

**Authors:** Zhihong Hu, Xuejia Zhao, Zhang Chen

**Affiliations:** ^1^ Gynaecology Section III, Shijiazhuang Maternal and Child Health Hospital Shijiazhuang China; ^2^ Faculty of Pharmaceutical Engineering University of N'djamena N'Djamena Chad

**Keywords:** anemia, patient prognosis, postoperative complications, preoperative management, surgical outcomes, total hip replacement

## Abstract

Anemia is a prevalent comorbidity among patients undergoing total hip replacement (THR) surgery, significantly affecting surgical outcomes and patient prognosis. This review synthesizes current literature on the relationship between anemia and THR, with a focus on postoperative complications, recovery times, and overall patient satisfaction. While several recent meta‐analyses have quantified the risks associated with anemia, our review offers a novel perspective by linking cellular mechanisms to clinical management strategies. We analyze various studies that highlight the prevalence of anemia in this patient population and its potential impact on surgical risks, including increased rates of transfusion, infection, and prolonged hospital stays. Furthermore, we explore the implications of anemia on functional recovery and long‐term outcomes, emphasizing the necessity for preoperative screening and management strategies. Our findings suggest that addressing anemia before THR may improve surgical outcomes and enhance patients' quality of life. This review underscores the importance of a multidisciplinary approach in the preoperative assessment and management of patients with anemia undergoing total hip replacement surgery.

## Graphical abstract

The processes involved in iron acquisition are closely controlled by hepcidin‐based regulatory mechanisms. Hepcidin is a peptide hormone mainly produced in the liver, acting as an acute‐phase reactant that responds to changes in plasma iron levels. It does this by binding to ferroportin, a protein responsible for exporting iron from cells, leading to its degradation. The expression of hepcidin rises when there are elevated levels of iron in the bloodstream and tissues, as well as during systemic inflammation or infections. Conversely, factors such as increased red blood cell production, iron deficiency, and low tissue oxygen levels can suppress its production, influenced by signals from the bone marrow, liver, and possibly muscle and fat cells. The rise in hepcidin levels triggered by inflammatory cytokines, particularly interleukin‐6, accounts for the sequestration of iron and the decreased availability of iron for erythropoiesis seen in anemia associated with chronic diseases.

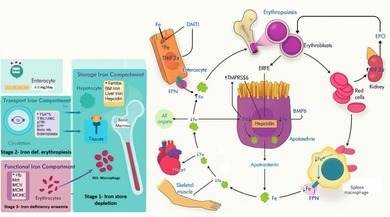

## 1 Introduction

Anemia is a prevalent condition characterized by a deficiency in the number or quality of red blood cells, leading to reduced oxygen transport throughout the body. Its significance in the context of total hip replacement (THR) surgery is multifaceted, impacting both preoperative and postoperative outcomes [1, 2]. THR, also known as total hip arthroplasty (THA), can be categorized into cemented and uncemented designs, each with distinct implications for implant stability and longevity. Cemented THR utilizes polymethylmethacrylate (PMMA) bone cement to bond the prosthesis to the host bone, providing immediate stability and allowing for early weight‐bearing [3, 4]. In THR, the selection of bearing surfaces is crucial for implant longevity and performance. Common options include metal‐on‐polyethylene (MoP), ceramic‐on‐polyethylene (CoP), and ceramic‐on‐ceramic (CoC) bearings, each with unique benefits and drawbacks [5, 6]. MoP is cost‐effective, while CoP and CoC provide better wear resistance and lower risk of osteolysis. Metal‐on‐metal (MoM) bearings, intended to improve durability, have been linked to serious complications such as metal ion release. Additionally, bimodular stem designs enhance adaptability to individual patient anatomies, optimizing THR outcomes [5, 6]. This technique is particularly beneficial in older patients with compromised bone quality, as the cement fills voids and enhances fixation. Preoperatively, anemia can increase the risk of complications during and after surgery, making it essential for healthcare providers to assess hemoglobin levels in patients. Identifying those at risk allows for the optimization of hemoglobin levels through interventions such as iron supplementation, erythropoiesis‐stimulating agents (ESAs), or blood transfusions. In clinical practice, anemia is often defined by hemoglobin levels below certain thresholds, which can vary based on guidelines or specific studies. For example, the World Health Organization (WHO) defines anemia as hemoglobin levels below 130 g/L for men and 120 g/L for women [7, 8]. Proactive management of anemia can reduce the likelihood of perioperative complications and may influence surgical decisions, as some surgeons may choose to delay THR until anemia is adequately addressed [9, 10]. Anemic patients are more susceptible to the effects of blood loss, which may require the implementation of blood conservation techniques and increase the likelihood of needing blood transfusions during or after the procedure. While transfusions can be necessary, they carry risks, including transfusion reactions and infections. Postoperatively, anemia can hinder recovery by diminishing exercise tolerance and overall energy levels, both critical for rehabilitation following THR [9, 10]. Patients with anemia may experience delayed mobilization and longer recovery times, complicating their overall health status. Additionally, evidence suggests that anemia is associated with an increased risk of postoperative infections, which can lead to further interventions and negatively impact recovery trajectories. Long‐term outcomes are also affected, as studies indicate that patients with anemia may experience poorer functional scores and higher rates of revision surgery after THR [11, 12]. In conclusion, the significance of anemia in THR is profound, influencing surgical risk, intraoperative management, and postoperative recovery. A comprehensive approach to managing anemia, including preoperative screening and optimization, is essential for improving surgical outcomes and enhancing the overall quality of care for patients undergoing THR. By addressing anemia proactively, healthcare providers can facilitate better recovery trajectories and reduce complications, ultimately contributing to more successful surgical interventions. This review underscores the importance of a multidisciplinary approach in the preoperative assessment and management of patients with anemia undergoing THR surgery.

## 2 Methodology

This review adopts a systematic approach to assess the impact of anemia on outcomes following THR surgery. The methodology consists of literature retrieval, study selection, data extraction, and synthesis. A comprehensive search strategy was designed to identify relevant studies published in peer‐reviewed journals from January 2000 to October 2025. The following electronic databases were utilized: PubMed, Cochrane Library, Scopus, and Web of Science. Search terms included “anemia,” “total hip replacement,” “surgical outcomes,” “postoperative complications,” and “patient prognosis.” Boolean operators (AND, OR) were employed to expand and refine the search results.

### 2.1 Prevalence of Anemia in THR Candidates

The prevalence of anemia among candidates for THR is a significant concern in orthopedic surgery, as it can influence surgical outcomes and recovery [13, 14]. Studies indicate that anemia affects a notable percentage of patients undergoing THR, with estimates ranging from 20% to 50%, depending on various demographic factors and underlying health conditions. This prevalence is not uniform and can vary significantly based on demographic factors and underlying health conditions. For instance, the prevalence may be around 20%–25% in primary THR cases, while it tends to be higher in high‐risk subgroups, reflecting the influence of factors such as age, comorbidities, and overall health status [15, 16]. Demographically, older adults are particularly susceptible to anemia, as age‐related physiological changes, chronic diseases, and nutritional deficiencies contribute to its development. Additionally, gender plays a role, with women often exhibiting higher rates of anemia due to factors such as menstruation, pregnancy, and iron deficiency [17, 18]. Furthermore, patients with comorbidities such as diabetes, chronic kidney disease (CKD), and inflammatory disorders are at an increased risk, as these conditions can impair red blood cell production or lead to chronic blood loss. Risk assessment for anemia in THR candidates is crucial for optimizing surgical outcomes [19]. Preoperative screening typically involves evaluating hemoglobin levels and conducting a thorough medical history to identify potential risk factors [20, 21]. This assessment allows healthcare providers to stratify patients based on their anemia status and tailor interventions accordingly [20, 21]. For instance, patients identified as anemic may benefit from preoperative treatments such as iron supplementation or ESAs to improve hemoglobin levels before surgery [22, 23]. Additionally, understanding the underlying causes of anemia—whether due to nutritional deficiencies, chronic disease, or other factors—enables a more targeted approach to management [22, 23]. By addressing anemia proactively, healthcare providers can mitigate the associated risks, such as increased likelihood of transfusion, longer hospital stays, and higher rates of postoperative complications. In summary, the prevalence of anemia among THR candidates is influenced by various demographic factors, including age, gender, and comorbidities. Effective risk assessment and management strategies are essential to optimize surgical outcomes and enhance recovery. Anemia significantly impacts surgical outcomes in patients undergoing THR, influencing various aspects of postoperative recovery and overall patient prognosis. One of the primary concerns is the increased risk of postoperative complications, including a higher incidence of infections, delayed wound healing, and cardiovascular events, which can lead to longer hospital stays and additional medical interventions [24, 25]. Anemic patients often experience slower recovery times due to reduced exercise tolerance and fatigue, resulting in prolonged rehabilitation and delayed return to daily activities [24, 25]. Furthermore, these patients may have poorer functional recovery scores, which can diminish their independence and quality of life postsurgery. The presence of anemia also raises the likelihood of requiring blood transfusions during or after the procedure, introducing additional risks such as transfusion‐related complications. Long‐term, anemia is associated with higher rates of revision surgery and an increased risk of chronic pain and disability, ultimately affecting patient satisfaction and leading to greater healthcare costs. Addressing anemia proactively is essential for optimizing surgical outcomes and enhancing the overall recovery experience for patients undergoing THR. To fully understand the implications of anemia on clinical outcomes following THR surgery, it is essential to explore the underlying biological mechanisms that contribute to these effects. Specifically, the role of iron biology is critical, as iron deficiency can exacerbate anemia and influence various physiological processes. By examining these mechanistic details, we can better appreciate how they relate to the observed clinical outcomes, thereby informing more effective management strategies for patients with anemia undergoing THR.

### 2.2 Impact of Anemia on Surgical Outcomes

Anemia significantly influences surgical outcomes, particularly, in patients undergoing procedures such as THR. One of the primary concerns associated with anemia is the increased risk of postoperative complications [25, 26]. Anemic patients often experience a higher incidence of infections, delayed wound healing, and cardiovascular events. The reduced oxygen‐carrying capacity of the blood can impair tissue oxygenation, which is critical for recovery and healing [25, 26]. Consequently, these complications can lead to a greater need for additional interventions, such as blood transfusions or extended medical management, further complicating the recovery process [27, 28]. In addition to complications, anemia also affects the length of hospital stay following surgery. Patients with anemia typically require more extended hospitalization due to the need for monitoring and management of their condition [27, 28]. The presence of anemia can lead to slower recovery times, as these patients may have reduced physical stamina and increased fatigue, hindering their ability to participate in rehabilitation activities [29, 30]. As a result, they may not meet discharge criteria as quickly as their nonanemic counterparts. This extended length of stay not only impacts patient well‐being but also places additional strain on healthcare resources, leading to increased healthcare costs [30]. In summary, anemia has a profound impact on surgical outcomes, contributing to a higher risk of postoperative complications and prolonging the length of hospital stays. Addressing anemia preoperatively can be crucial in improving recovery trajectories and minimizing the associated risks, ultimately enhancing the overall quality of care for patients undergoing surgical procedures. Transfusion requirements in the context of surgical procedures, particularly THR, are a critical consideration for both patient safety and surgical outcomes [31, 32]. Blood transfusions may be necessary for patients who experience significant blood loss during surgery or who present with anemia before the procedure [31, 32]. The decision to administer a transfusion is influenced by several factors, including the patient's preoperative hemoglobin levels, the anticipated blood loss during surgery, and the overall health status of the patient [33, 34]. In patients undergoing THR, the potential for blood loss can be substantial due to the nature of the procedure, which involves extensive manipulation of bone and soft tissue [33, 34]. Anemic patients are at a higher risk of requiring transfusions, as their baseline hemoglobin levels may already be low, making them more susceptible to the effects of intraoperative blood loss [35, 36]. Transfusions can help restore adequate hemoglobin levels, improve oxygen delivery to tissues, and support recovery. However, the use of blood transfusions is not without risks. Transfusions can lead to complications such as allergic reactions, transfusion‐related acute lung injury (TRALI), and infections [35, 36]. Additionally, there is evidence suggesting that unnecessary transfusions may be associated with poorer outcomes, including increased rates of postoperative infections and longer hospital stays [37, 38]. Therefore, it is essential for healthcare providers to carefully assess the need for transfusions and consider alternatives, such as blood conservation techniques, autologous blood donation, or the use of ESAs to boost red blood cell production preoperatively. In summary, transfusion requirements in THR patients are a vital aspect of perioperative care [37, 38]. A thorough understanding of the factors influencing the need for transfusions, along with a careful assessment of risks and benefits, is essential for optimizing patient outcomes and ensuring safe surgical practices. By managing blood loss effectively and considering alternatives to allogeneic transfusions, healthcare providers can enhance recovery and minimize complications associated with surgical procedures. By recognizing and addressing anemia in this patient population, healthcare providers can improve overall surgical success and patient quality of life (Table 1).

**TABLE 1:** Anemia and its influence on surgical outcomes in THR patients.

Outcome category	Impact of anemia	Potential consequences
Postoperative complications	– Increased risk of infections (e.g., surgical site infections). – Higher incidence of delayed wound healing. – Greater likelihood of cardiovascular events (e.g., myocardial infarction).	– Longer hospital stays and additional interventions. – Increased healthcare costs and resource utilization. – Potential for increased morbidity and mortality.
Length of hospital stay	– Anemic patients often require longer hospitalization due to complications.	– Delayed discharge and increased burden on healthcare facilities.
Recovery time	– Slower recovery due to reduced exercise tolerance and fatigue.	– Prolonged rehabilitation and delayed return to daily activities.
Functional outcomes	– Poorer functional recovery scores (e.g., mobility, strength).	– Decreased independence and quality of life postsurgery.
Transfusion requirements	– Higher likelihood of requiring blood transfusions during or after surgery.	– Increased risk of transfusion‐related complications (e.g., reactions, infections).
Long‐term prognosis	– Anemia associated with higher rates of revision surgery. – Increased risk of chronic pain and disability.	– Potential for decreased long‐term satisfaction and quality of life. – Long‐term healthcare costs and impact on patient well‐being.

### 2.3 Cellular Aspects of Iron on Bone Healing

**FIGURE 1:**
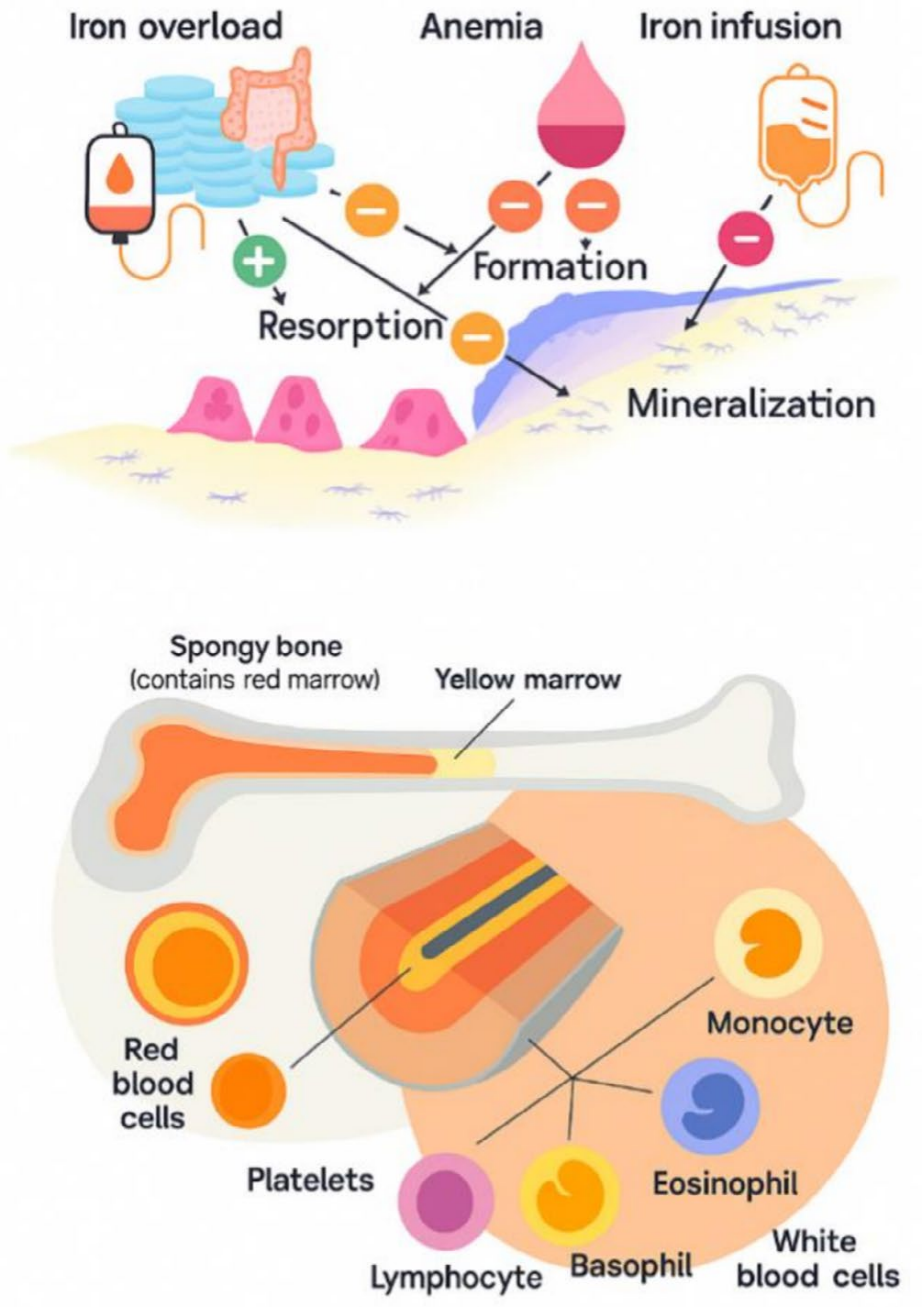
Effects of iron overload, deficiency, and anemia treatments on bone.

Bone healing is a complex biological process that involves a series of cellular and molecular events, including inflammation, cell proliferation, and tissue remodeling [39]. Among the various factors that influence bone healing, iron plays a crucial role due to its involvement in several cellular functions, including oxygen transport, collagen synthesis, and the regulation of various enzymatic reactions [39]. Understanding the cellular aspects of iron in bone healing can provide insights into potential therapeutic strategies for enhancing recovery from bone injuries. Iron is an essential micronutrient that is primarily stored in the body as ferritin and is transported in the bloodstream bound to transferrin [40]. During the bone healing process, iron is critical for the proliferation and differentiation of osteoblasts, the cells responsible for new bone formation. Osteoblasts require iron for the synthesis of collagen, which is a major component of the bone extracellular matrix [41, 42]. Collagen provides structural integrity to the bone and serves as a scaffold for mineral deposition. Additionally, iron is involved in the activity of prolyl hydroxylase, an enzyme that stabilizes collagen by hydroxylating proline residues, thereby enhancing the mechanical properties of the newly formed bone. Moreover, iron plays a significant role in the inflammatory phase of bone healing [41, 42]. Macrophages, which are key players in the inflammatory response, utilize iron to produce reactive oxygen species (ROS) that help eliminate pathogens and debris at the injury site. However, excessive iron accumulation can lead to oxidative stress, which may hinder the healing process. Therefore, maintaining an optimal balance of iron is essential for effective bone healing [43]. The interplay between iron and other cellular components is also noteworthy. For instance, the presence of hypoxia‐inducible factors (HIFs) in the hypoxic environment of a fracture site can stimulate the expression of genes involved in iron metabolism [44]. HIFs promote the expression of transferrin and ferritin, enhancing iron availability for osteoblasts and other cells involved in bone repair [44]. Additionally, the role of iron in angiogenesis, the formation of new blood vessels, is critical for supplying oxygen and nutrients to the healing bone. Endothelial cells, which line blood vessels, also require iron for their proliferation and function, further linking iron metabolism to the overall healing process (Figure 1).

**FIGURE 2:**
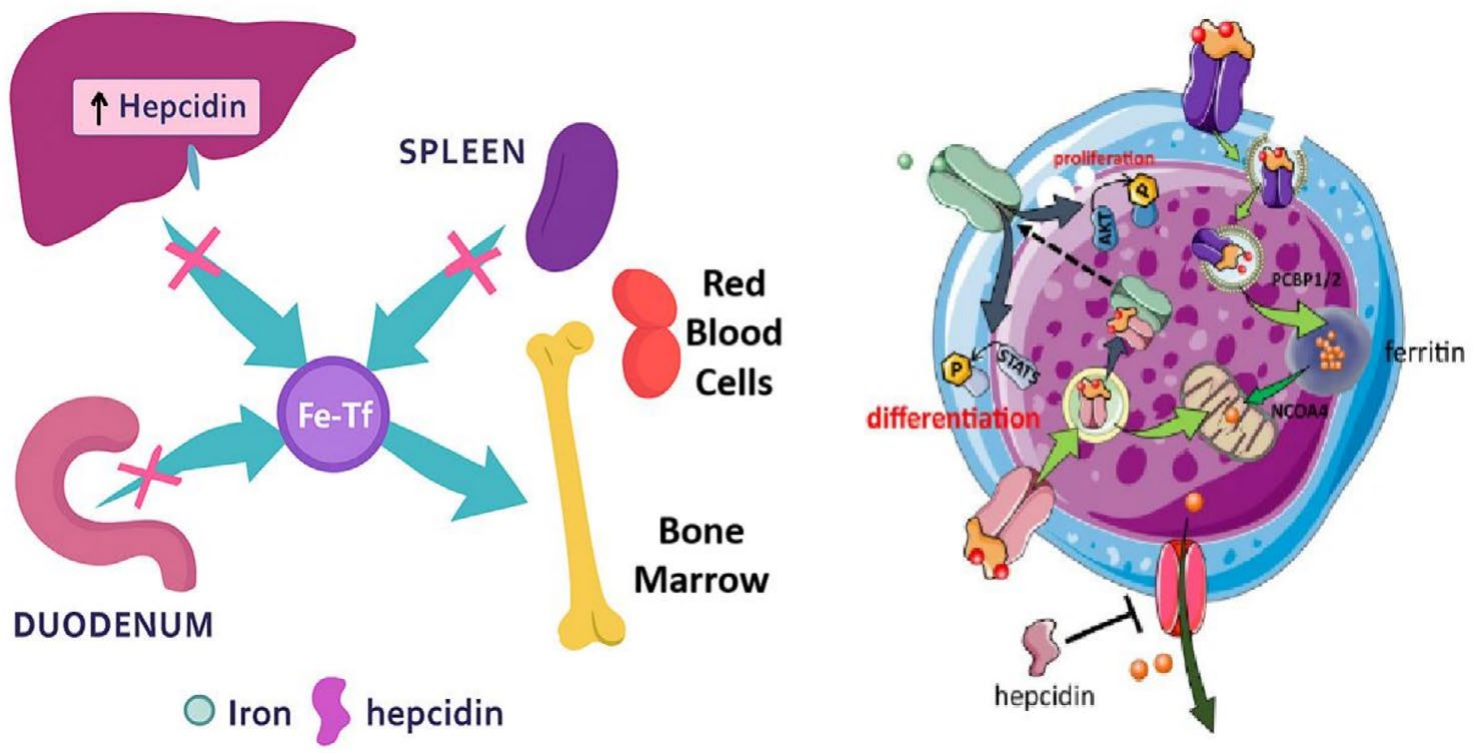
Cellular aspects of iron on bone healing.

In sum, iron is a vital element in the cellular aspects of bone healing, influencing osteoblast function, collagen synthesis, and the inflammatory response. The balance of iron is crucial; while it is necessary for various cellular processes, excessive iron can lead to oxidative stress and impede healing. Future research should focus on elucidating the precise mechanisms by which iron affects bone healing and exploring potential therapeutic interventions that can optimize iron levels to enhance recovery from bone injuries. Understanding these cellular dynamics will pave the way for improved clinical outcomes in patients with bone fractures and other related conditions (Figure 2).

Iron overload, deficiency, and anemia treatments can significantly impact bone health and healing. Iron overload, often seen in conditions like hemochromatosis or repeated blood transfusions, can lead to oxidative stress and inflammation, which may impair osteoblast function and promote bone resorption, resulting in decreased bone density and increased fracture risk [45, 46]. Conversely, iron deficiency, commonly associated with anemia, can hinder collagen synthesis and osteoblast proliferation, negatively affecting bone formation and repair. Treatments for anemia, such as iron supplementation, can improve hemoglobin levels and enhance oxygen delivery to tissues, potentially benefiting bone healing; however, excessive supplementation must be carefully monitored to avoid the adverse effects of iron overload [45, 46]. Thus, maintaining an optimal balance of iron is crucial for preserving bone health, as both deficiency and excess can disrupt the delicate processes involved in bone remodeling and regeneration.

### 2.4 Erythropoiesis Suppression

Anemia, characterized by a deficiency in the number or quality of red blood cells (RBCs), is a prevalent condition that can arise from various underlying causes, including nutritional deficiencies, chronic diseases, and bone marrow disorders [47, 48]. One of the critical aspects of anemia is its relationship with erythropoiesis suppression, a process where the production of red blood cells in the bone marrow is inhibited [47, 48]. Understanding the interplay between anemia and erythropoiesis suppression is essential for developing effective treatment strategies and improving patient outcomes. Erythropoiesis, the process of red blood cell formation, is primarily regulated by erythropoietin (EPO), a hormone produced by the kidneys in response to low oxygen levels in the blood [49]. In healthy individuals, EPO stimulates the bone marrow to increase the production of erythroid progenitor cells, which differentiate into mature RBCs. However, in conditions such as CKD, the production of EPO is often impaired, leading to decreased erythropoiesis and subsequent anemia [50]. This form of anemia, known as anemia of chronic disease, is characterized by the suppression of erythropoiesis due to inflammatory cytokines that inhibit erythroid progenitor cell proliferation and function [50]. Moreover, iron deficiency is a common contributor to anemia and can further exacerbate erythropoiesis suppression. Iron is a crucial component of hemoglobin, the protein responsible for oxygen transport in RBCs [51]. When iron levels are insufficient, the bone marrow's ability to produce functional red blood cells is compromised, leading to microcytic anemia. In such cases, even with adequate EPO levels, the lack of iron can prevent effective erythropoiesis, creating a cycle of anemia and suppressed red blood cell production. In addition to nutritional deficiencies, certain medications and treatments can also lead to erythropoiesis suppression [52]. For instance, chemotherapy agents used in cancer treatment can damage the bone marrow, resulting in reduced RBC production and subsequent anemia. Similarly, autoimmune disorders may trigger the production of antibodies against erythroid progenitor cells, further inhibiting erythropoiesis [53]. In these scenarios, addressing the underlying cause of anemia is crucial for restoring normal erythropoiesis and improving hemoglobin levels [53]. Treatment strategies for anemia often focus on correcting the underlying causes while stimulating erythropoiesis. In cases of iron deficiency anemia, iron supplementation can enhance hemoglobin synthesis and promote red blood cell production. For anemia associated with CKD, ESAs may be administered to boost EPO levels and stimulate the bone marrow. However, careful monitoring is essential, as excessive stimulation of erythropoiesis can lead to complications, including hypertension and increased risk of thromboembolic events [54]. Finally, anemia and erythropoiesis suppression are intricately linked, with various factors contributing to the development and persistence of anemia. Understanding the mechanisms underlying erythropoiesis suppression is vital for effective diagnosis and treatment [54]. By addressing both the anemia and its root causes, healthcare providers can improve erythropoiesis, enhance patient quality of life, and reduce the morbidity associated with this common condition. Continued research into the complex interactions between anemia, erythropoiesis, and underlying health conditions will be essential for advancing treatment approaches and improving patient outcomes.

### 2.5 Iron‐Deficiency‐Induced Hypoxia in Bone Homeostasis

Iron deficiency is a common nutritional disorder that can lead to hypoxia, a condition characterized by insufficient oxygen availability in tissues, which significantly impacts bone homeostasis [55]. Iron is essential for the production of hemoglobin, the protein responsible for oxygen transport in red blood cells. When iron levels are low, the body struggles to produce functional hemoglobin, resulting in reduced oxygen delivery to bone tissue. This hypoxic environment activates HIFs, which regulate genes involved in various processes critical for bone health [55]. Under hypoxic conditions, osteoblast function is impaired, leading to decreased bone formation, while the activity of osteoclasts may be stimulated, resulting in increased bone resorption [56]. This imbalance between bone formation and resorption can compromise skeletal integrity and contribute to bone loss. Addressing iron deficiency is crucial for restoring normal bone homeostasis and mitigating the adverse effects of hypoxia on bone health. Iron supplementation can enhance hemoglobin levels, improve oxygen delivery to tissues, and alleviate the hypoxic state, thereby supporting osteoblast function and promoting bone formation [57]. Additionally, interventions that promote angiogenesis and improve blood flow to bone tissue may further enhance skeletal health in individuals with iron deficiency. Understanding the intricate relationship between iron metabolism, hypoxia, and bone homeostasis is essential for developing targeted therapeutic strategies to preserve bone integrity and improve the quality of life for affected individuals. Continued research in this area will be vital for advancing our knowledge of skeletal health and informing effective interventions.

### 2.6 Iron in Collagen Synthesis

**FIGURE 3:**
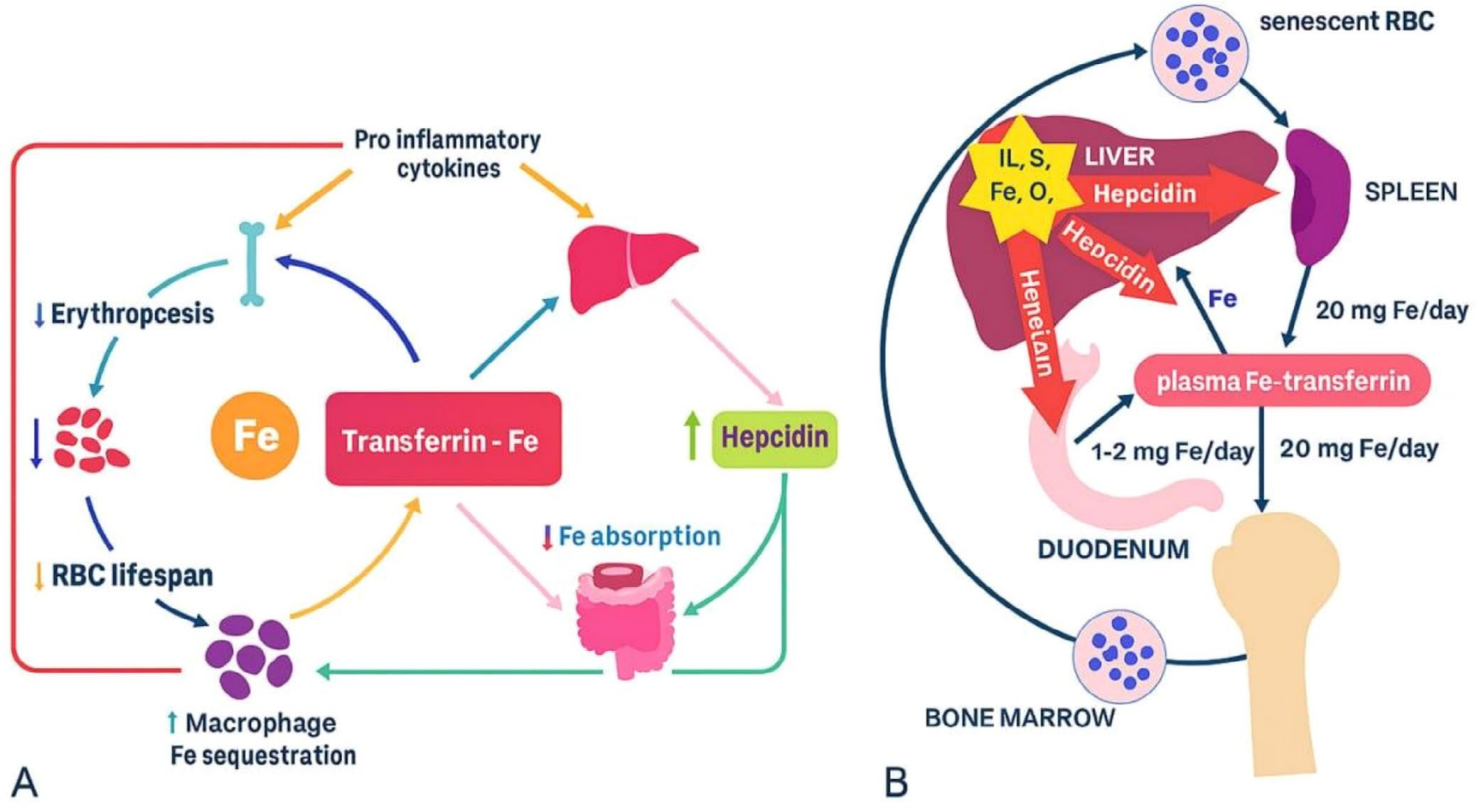
(A) Changes in iron recycling during anemia of inflammation involve several key processes. Normally, transferrin‐bound iron circulates to the bone marrow to aid in the production of red blood cells (RBCs). When RBCs reach the end of their lifespan, they are consumed by macrophages, and the iron is recycled back into circulation to support new erythropoiesis. Additionally, dietary iron can enter the bloodstream from the duodenum based on the body's requirements. However, in anemia of inflammation, increased levels of proinflammatory cytokines hinder erythropoiesis in the bone marrow and reduce RBC lifespan due to heightened macrophage activity and the engulfing of RBCs (erythrophagocytosis). Cytokines such as IL‐6 and IL‐1β prompt the liver to enhance the production of hepcidin antimicrobial peptide (HAMP). Hepcidin restricts iron release from reticuloendothelial system cells, including enterocytes and macrophages, by binding to and promoting the degradation of ferroportin, the iron exporter. This leads to decreased iron absorption and greater iron storage within macrophages. As a result, the combination of reduced erythropoiesis, shortened RBC lifespan, iron storage in macrophages, and lower iron absorption disrupts iron recycling and leads to inadequate iron availability for erythropoiesis and hemoglobin production, ultimately causing anemia. (B) The function of hepcidin in iron regulation is crucial. Hepcidin controls the absorption of iron in the intestines, the recycling of iron by macrophages, and the release of iron from liver reserves. Additionally, the secretion of hepcidin is influenced by the levels of iron stored in the body, oxygen availability, and inflammatory signals, particularly interleukin‐6 (IL‐6). RBC refers to red blood cells.

Iron plays a crucial role in collagen synthesis, a vital process for maintaining the structural integrity of connective tissues in the body. Collagen, the most abundant protein in mammals, provides strength and elasticity to skin, tendons, ligaments, and bones [58]. Iron is a key cofactor for prolyl and lysyl hydroxylase enzymes, which are essential for the post‐translational modifications of collagen [58]. These enzymes facilitate the hydroxylation of proline and lysine residues, which is critical for the stability and proper formation of the collagen triple helix. Without adequate iron levels, the activity of these enzymes can be compromised, leading to impaired collagen synthesis and, consequently, weakened connective tissues [59]. Moreover, iron deficiency can have broader implications for overall health, as collagen is not only important for structural support but also plays a role in wound healing and tissue repair. Insufficient collagen production due to low iron levels can result in delayed healing processes and increased susceptibility to injuries [55, 60]. Additionally, conditions such as anemia, which is characterized by low iron levels, can further exacerbate the effects of impaired collagen synthesis, leading to symptoms like brittle nails, hair loss, and skin issues. Therefore, maintaining adequate iron levels is essential not only for optimal collagen synthesis but also for overall tissue health and resilience. In addition to its enzymatic roles, iron also influences collagen synthesis through its involvement in cellular metabolism and signaling pathways [61]. Iron is a critical component of various proteins and enzymes that facilitate cellular respiration and energy production, which are essential for the proliferation and function of fibroblasts—the cells responsible for producing collagen. When iron levels are adequate, fibroblasts can efficiently generate the energy required for collagen synthesis and secretion. Furthermore, iron acts as a signaling molecule that can modulate the expression of genes involved in collagen production [62]. For instance, iron‐responsive proteins can regulate the transcription of collagen genes, ensuring that collagen synthesis is appropriately adjusted in response to the body's needs. This intricate interplay between iron availability and collagen production underscores the importance of maintaining optimal iron levels not only for structural integrity but also for the dynamic regulation of tissue repair and remodeling processes. Consequently, understanding the relationship between iron and collagen synthesis can provide valuable insights into the management of conditions associated with connective tissue disorders, wound healing, and overall musculoskeletal health (Figure 3).

## 3 Iron in Vitamin D Metabolism

Iron plays a significant role in the metabolism of vitamin D, a crucial nutrient for maintaining bone health and regulating calcium levels in the body. Vitamin D undergoes a series of transformations in the liver and kidneys to become its active form, calcitriol [63]. During these metabolic processes, iron is essential for the activity of various enzymes involved in the hydroxylation of vitamin D. Specifically, the enzyme 25‐hydroxylase, which converts vitamin D into its primary circulating form, relies on iron as a cofactor. Without sufficient iron, the efficiency of this enzymatic conversion can be compromised, leading to lower levels of active vitamin D in the body [63]. This deficiency can have cascading effects on calcium homeostasis and bone mineralization, potentially resulting in conditions such as osteomalacia or osteoporosis. Moreover, the interplay between iron and vitamin D metabolism extends beyond enzymatic activity; it also involves the regulation of gene expression related to vitamin D receptors [64]. Iron deficiency has been shown to affect the expression of receptors that are crucial for the cellular response to vitamin D. When iron levels are insufficient, the body's ability to respond effectively to vitamin D may be compromised, potentially exacerbating issues related to bone health and calcium absorption. Furthermore, emerging research indicates that the interplay between iron and vitamin D may have significant implications for immune function, as both nutrients are involved in the modulation of immune responses [65]. Consequently, maintaining adequate iron levels is essential not only for optimal vitamin D metabolism but also for overall health. This underscores the importance of a balanced diet that supports the interaction of these vital nutrients. A comprehensive understanding of this relationship can inform strategies aimed at preventing and managing deficiencies that may adversely affect bone health and immune function.

## 4 Effect of Iron Deficiency on Osteoclast and Osteoblast Differentiation and Function

Iron deficiency is a widespread nutritional issue that can have significant implications for bone health, particularly through its effects on the differentiation and function of osteoclasts and osteoblasts. Osteoclasts are specialized cells responsible for bone resorption, while osteoblasts are involved in bone formation [66]. The balance between these two cell types is crucial for maintaining bone homeostasis, and disruptions in their function can lead to conditions such as osteoporosis and increased fracture risk [66]. Iron plays a vital role in the differentiation and activity of both osteoclasts and osteoblasts. In osteoblasts, iron is essential for various cellular processes, including collagen synthesis and mineralization, which are critical for bone formation. Iron deficiency can impair the proliferation and differentiation of osteoblasts, leading to reduced bone formation [67]. This impairment occurs because iron is a cofactor for enzymes involved in collagen synthesis, and its deficiency can result in decreased collagen production, ultimately compromising the structural integrity of the bone matrix [67]. Additionally, iron deficiency can activate stress response pathways that further inhibit osteoblast function, exacerbating the negative impact on bone health. On the other hand, iron deficiency can also influence osteoclast differentiation and activity [68]. Osteoclasts originate from monocyte/macrophage precursors, and their differentiation is regulated by various factors, including receptor activator of nuclear factor kappa‐Β ligand (RANKL). Studies have shown that iron deficiency can enhance the expression of RANKL, promoting osteoclastogenesis and leading to increased bone resorption [69]. This imbalance, characterized by heightened osteoclast activity coupled with diminished osteoblast function, can result in a net loss of bone mass and density. Furthermore, the inflammatory response associated with iron deficiency may also contribute to increased osteoclast activity, further disrupting the delicate balance between bone formation and resorption [69]. In summary, iron deficiency adversely affects both osteoclast and osteoblast differentiation and function, leading to an imbalance that can compromise bone health. The impairment of osteoblast function due to reduced collagen synthesis and mineralization, combined with the promotion of osteoclastogenesis, results in increased bone resorption and decreased bone formation. Addressing iron deficiency through dietary changes or supplementation may help restore the balance between these two cell types, ultimately supporting bone health and reducing the risk of osteoporosis and fractures. Understanding the mechanisms by which iron influences bone cell function is essential for developing targeted interventions to mitigate the effects of iron deficiency on skeletal health.

### 4.1 Anemia and Functional Recovery

Anemia can significantly impact functional recovery in patients undergoing THR, presenting various rehabilitation challenges and influencing long‐term outcomes and quality of life [70, 71]. Following THR, patients typically engage in a structured rehabilitation program aimed at restoring mobility, strength, and overall function. However, the presence of anemia can hinder this process [70, 71]. Anemic patients often experience fatigue, reduced exercise tolerance, and decreased stamina, making it more difficult for them to participate fully in rehabilitation activities [72, 73]. This can lead to slower progress in regaining strength and mobility, ultimately prolonging the recovery period. The challenges posed by anemia during rehabilitation can also affect the psychological well‐being of patients [72, 73]. As they struggle to meet rehabilitation goals, feelings of frustration and discouragement may arise, further impacting their motivation to engage in physical therapy. Additionally, the need for frequent monitoring and potential medical interventions related to anemia can disrupt the continuity of rehabilitation, leading to inconsistent participation and suboptimal recovery outcomes [74, 75]. In terms of long‐term outcomes, anemia can have lasting effects on a patient's quality of life following THR. Studies have shown that patients with anemia may experience poorer functional outcomes, including reduced mobility and lower levels of physical activity compared to their non‐anemic counterparts [74, 75]. This decline in physical function can contribute to a decreased ability to perform daily activities, ultimately affecting overall independence and quality of life. Furthermore, the association between anemia and increased rates of postoperative complications can lead to additional health issues, further complicating recovery and diminishing life satisfaction [13, 76]. In conclusion, anemia poses significant challenges to functional recovery in patients undergoing THR. The impact on rehabilitation efforts, combined with the potential for poorer long‐term outcomes and diminished quality of life, underscores the importance of addressing anemia proactively [13, 76]. By optimizing hemoglobin levels and managing anemia effectively before and after surgery, healthcare providers can enhance rehabilitation success, improve functional recovery, and ultimately contribute to a better quality of life for patients.

### 4.2 Management Strategies for Anemia in Preoperative Patients

Effective management of anemia in preoperative patients undergoing THR is crucial for optimizing surgical outcomes and enhancing recovery. The management process typically involves two key components: screening and diagnosis, followed by appropriate treatment options and protocols.

### 4.3 Screening and Diagnosis

The first step in managing anemia is thorough screening and diagnosis. Preoperative assessment should include a comprehensive evaluation of the patient's medical history, physical examination, and laboratory tests to determine hemoglobin levels and identify the presence of anemia [77, 78]. Common laboratory tests include complete blood counts (CBC) and iron studies, which help to assess not only hemoglobin levels but also the underlying causes of anemia, such as iron deficiency, chronic disease, or vitamin deficiencies [77, 78]. Identifying the type and cause of anemia is essential, as it informs the subsequent treatment approach. Additionally, risk factors such as age, gender, and comorbidities should be considered, as these can influence the likelihood of anemia and its potential impact on surgical outcomes.

### 4.4 Treatment Options and Protocols

Once anemia is diagnosed, appropriate treatment options must be implemented. The choice of treatment depends on the underlying cause of anemia and the severity of the condition [79, 80]. For patients with iron deficiency anemia, oral iron supplements or intravenous iron therapy may be prescribed to replenish iron stores and improve hemoglobin levels. In cases where anemia is due to chronic disease or other factors, ESAs may be considered to stimulate red blood cell production [79, 80]. In addition to pharmacological interventions, nutritional counseling can play a vital role in managing anemia. Patients may benefit from dietary modifications to increase the intake of iron‐rich foods, such as red meat, leafy greens, and fortified cereals, as well as foods high in vitamin C to enhance iron absorption [81, 82]. Protocols for managing anemia in preoperative patients should also include regular monitoring of hemoglobin levels and overall response to treatment. This ongoing assessment allows healthcare providers to adjust treatment plans as necessary and ensure that patients reach optimal hemoglobin levels before surgery [81, 82]. In some cases, if anemia is severe or unresponsive to medical management, blood transfusions may be considered as a last resort to stabilize the patient before surgery. In summary, the management of anemia in preoperative patients undergoing THR involves systematic screening and diagnosis, followed by targeted treatment options and protocols. By addressing anemia proactively, healthcare providers can improve surgical outcomes, reduce the risk of complications, and enhance the overall recovery experience for patients (Table 2).

**TABLE 2:** Management protocols for anemia in preoperative THR patients.

Step	Description	References
Screening	– Conduct a comprehensive medical history and physical examination. – Perform laboratory tests, including complete blood count (CBC) and iron studies. – Identify risk factors (age, gender, comorbidities) associated with anemia.	[83, 84, 85]
Diagnosis	– Determine hemoglobin levels to confirm anemia. – Identify the type and cause of anemia (e.g., iron deficiency, chronic disease).	[86, 87, 88]
Treatment options	Iron deficiency anemia: – Oral iron supplements or intravenous iron therapy. Anemia due to chronic disease: – Consider ESAs to stimulate red blood cell production. Nutritional counseling: – Advise on dietary modifications to increase iron intake (e.g., red meat, leafy greens). – Recommend foods high in vitamin C to enhance iron absorption.	[29, 89, 90]
Monitoring	– Regularly monitor hemoglobin levels and response to treatment. – Adjust treatment plans as necessary to achieve optimal hemoglobin levels before surgery.	[70, 91, 92]
Consideration of transfusions	– If anemia is severe or unresponsive to treatment, consider blood transfusions as a last resort.	[36, 93]
Preoperative optimization	– Ensure that hemoglobin levels are optimized before surgery to minimize risks.	[94, 95]

Adequate management of perioperative anemia is one of the fundamental pillars of patient blood management (PBM). Several consensus documents and clinical practice guidelines developed by national and international working groups and scientific societies advocate for a systematic approach to treating preoperative anemia. These guidelines emphasize the importance of early identification and intervention to optimize hemoglobin levels before surgery. Regarding postoperative anemia, most recommendations suggest employing a restrictive threshold for packed red blood cell transfusion (PRBCT) in cases of severe anemia. However, there is a notable lack of guidance on pharmacological treatments for postoperative anemia, leading to variability in clinical practice. This inconsistency highlights the need for more comprehensive recommendations to standardize care and improve outcomes for patients experiencing anemia in the perioperative setting.

## 5 Future Directions

As the understanding of anemia in patients undergoing THR continues to evolve, several future directions for research and clinical practice emerge. One critical area for further research is the exploration of the underlying mechanisms of anemia in the context of orthopedic surgery. Investigating the specific causes of anemia in THR patients, including the roles of nutritional deficiencies, chronic diseases, and inflammatory processes, can lead to more targeted and effective treatment strategies. Additionally, studies examining the impact of preoperative anemia management on postoperative outcomes, such as recovery times, functional mobility, and quality of life, are essential. Longitudinal studies that track patients over time can provide valuable insights into the long‐term effects of anemia and its management on overall health and well‐being. Another important area for research is the development and evaluation of standardized protocols for anemia screening and management in surgical patients. Establishing evidence‐based guidelines can help ensure consistency in care and improve outcomes across different healthcare settings. To enhance this section, a proposed flow chart outlining a clinical algorithm for the detection, treatment, and management of anemia in patients scheduled for THR could be included. This algorithm would serve as a practical tool for healthcare providers, guiding them through the steps of screening, diagnosing, and implementing appropriate interventions based on the severity of anemia. Furthermore, investigating the cost‐effectiveness of various anemia management strategies, including the use of iron supplementation versus blood transfusions, can inform clinical decision‐making and resource allocation. The management of anemia in THR patients underscores the importance of a multidisciplinary approach to care. Collaboration among orthopedic surgeons, hematologists, primary care physicians, nutritionists, and rehabilitation specialists is essential for optimizing patient outcomes. Each discipline brings unique expertise that can contribute to a comprehensive understanding of anemia and its implications for surgical recovery. For instance, nutritionists can provide guidance on dietary modifications to enhance iron intake, while rehabilitation specialists can develop tailored exercise programs that accommodate the limitations posed by anemia. Moreover, effective communication and coordination among healthcare providers are crucial for ensuring that patients receive timely and appropriate interventions. Implementing multidisciplinary care pathways can facilitate this collaboration, allowing for seamless transitions between different stages of care and improving overall patient management. In summary, future directions in the study and management of anemia in THR patients should focus on understanding its underlying causes, developing standardized protocols, and fostering multidisciplinary collaboration. By addressing these areas and incorporating a clinical algorithm for anemia management, healthcare providers can enhance the quality of care, improve surgical outcomes, and ultimately contribute to better health and quality of life for patients undergoing THR. Proposed table (Table 3) outlining a clinical algorithm for the detection, treatment, and management of anemia in patients scheduled for THA.

**TABLE 3:** Outlining a clinical algorithm for the detection, treatment, and management of anemia in patients scheduled for THA.

Step	Action	Outcome
Preoperative screening	– Assess patient history (chronic diseases, nutritional status) – Conduct laboratory tests (CBC, iron studies)	– Identify potential risk factors for anemia. – Establish baseline hemoglobin and iron status.
Determine anemia status	Evaluate hemoglobin level: – Normal (Hb > 12 g/dL) – Mild anemia (Hb 10–12 g/dL) – Moderate to severe anemia (Hb < 10 g/dL)	– Proceed with THA. – Monitor and consider preoperative optimization. – Initiate further evaluation and management.
Identify underlying causes	– Assess for nutritional deficiencies (iron, vitamin B12, folate) – Evaluate for chronic diseases (e.g., chronic kidney disease, inflammatory disorders) – Consider other factors (blood loss history, medications)	– Determine specific causes of anemia. – Tailor management based on underlying causes. – Comprehensive understanding of anemia etiology.
Management strategies	For nutritional deficiencies: – Iron Supplementation (oral or intravenous) – Vitamin B12 or folate supplementation as needed For chronic disease‐related anemia: – Treat underlying condition – Consider erythropoiesis‐stimulating agents if appropriate For severe anemia: – Blood transfusion may be necessary	– Improve hemoglobin levels. – Address specific deficiencies. – Optimize overall health. – Enhance red blood cell production. – Rapidly increase hemoglobin levels if critically low.
Reassess hemoglobin levels	– Postmanagement testing (repeat CBC) – If hemoglobin improves: – If hemoglobin does not improve:	– Evaluate response to treatment. – Proceed with THA. – Reevaluate management plan and consider referral to a specialist.
Multidisciplinary collaboration	– Involve orthopedic surgeons, hematologists, nutritionists, and rehabilitation specialists	– Ensure comprehensive care and optimize patient outcomes.
Postoperative monitoring	– Monitor hemoglobin levels and overall recovery	– Address any complications related to anemia management.

## 6 Conclusion

The management of anemia in patients undergoing THR is a critical component of preoperative care that significantly impacts surgical outcomes and recovery. The notable prevalence of anemia in this population, influenced by various demographic factors and comorbidities, is associated with increased postoperative complications, longer hospital stays, and challenges in rehabilitation. To optimize patient care, healthcare providers must implement comprehensive screening protocols to identify anemia early in the preoperative phase, followed by tailored treatment strategies addressing its underlying causes, such as iron supplementation and nutritional counseling. A multidisciplinary approach involving orthopedic surgeons, hematologists, and nutritionists is essential for delivering individualized care. However, this study has limitations, including a potential selection bias in the patient population and a lack of long‐term follow‐up data to assess the sustained impact of anemia management on functional outcomes. Looking ahead, there is a pressing need for clinical trials focused on the correction of anemia and its effects on long‐term functional outcomes in patients undergoing THR. By advancing our understanding in this area, we can further enhance surgical outcomes and improve the quality of life for patients.

## Author Contributions

**Zhihong Hu, Xuejia Zhao** and **Zhang Chen:** conceptualization and supervision, investigation; data curation; writing – original draft.

## Ethics Statement

The authors have nothing to report.

## Consent

The authors have nothing to report.

## Conflicts of Interest

The authors declare no conflicts of interest.

## Data Availability

Data sharing not applicable to this article as no datasets were generated or analysed during the current study.
